# Does Interrater Variation Influence the Classification According to the Diagnostic Criteria for Multiple Sclerosis?

**DOI:** 10.1111/ene.70469

**Published:** 2026-01-23

**Authors:** Nima Mahmoudi, Julius Renne, Franz Felix Konen, Konstantin Fritz Jendretzky, Nora Möhn, Lea Grote‐Levi, Stefan Gingele, Martin W. Hümmert, Philipp Schwenkenbecher, Alexander Soldatov, Kurt‐Wolfram Sühs, Peter Raab, Thomas Skripuletz, Mike P. Wattjes

**Affiliations:** ^1^ Department of Neuroradiology Charité ‐ Universitätsmedizin Berlin, Corporate Member of Freie Universität Berlin, Humboldt‐Universität zu Berlin Berlin Germany; ^2^ Department of Diagnostic and Interventional Neuroradiology Hannover Medical School Hannover Germany; ^3^ Department of Neurology Hannover Medical School Hannover Germany

**Keywords:** demyelination, McDonald criteria, MR imaging, multiple sclerosis, optic nerve, optic nerve imaging

## Abstract

**Background:**

Studies on interrater agreement in MRI analysis for patients experiencing a first clinical episode suggestive of multiple sclerosis (MS) are limited and outdated. This study aimed to evaluate interrater agreement for lesion identification in the brain, optic nerve, and spinal cord, and to assess variability in applying the 2017 and 2024 McDonald criteria.

**Methods:**

Seventy‐eight patients underwent a standardized multisequence 3 Tesla MRI of the brain, optic nerves, and spinal cord, analyzed by three readers with varying expertise. Lesions were categorized based on location according to McDonald criteria. Interrater variability was measured using Cohen's κ for pairwise reader comparisons and Fleiss κ for overall agreement.

**Results:**

Interrater agreement ranged from slight to fair for total lesion count and moderate to substantial for lesion presence. The highest agreement occurred for spinal cord lesions (κ = 0.84), periventricular T2 lesions (κ = 0.70), and gadolinium‐enhancing brain lesions (κ = 0.75). Classification agreement based on diagnostic criteria was substantial between the 2017 and 2024 McDonald criteria revisions (κ = 0.65).

**Conclusions:**

Despite advanced standardized imaging protocols at 3 T, interrater agreement regarding lesion counts does not improve substantially. However, agreement in classifying lesions according to diagnostic criteria is consistent between the 2017 and 2024 criteria.

## Graphical abstract

This study investigates interrater variability in MRI‐based lesion detection and its impact on multiple sclerosis diagnosis in accordance with the 2017 and 2024 McDonald criteria. Despite differences among raters, diagnostic agreement between both criteria versions remained high. The results show that diagnostic consistency is strong and that standardized MRI interpretation is important.

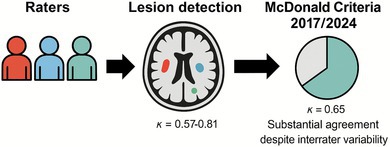

## 1 Introduction

Magnetic resonance imaging (MRI) of the brain and the spinal cord is the most sensitive paraclinical method with respect to the detection of focal inflammatory demyelinating lesions and has been incorporated into the first version of the McDonald diagnostic criteria and the subsequent revisions facilitating the demonstration of lesion dissemination in space (DIS) and in time (DIT) [1, 2, 3, 4, 5]. Regarding the DIS criteria, the demonstration of one or more lesions in at least two certain anatomic locations such as the cortical gray matter and/or juxtacortical white matter, periventricular white matter, infratentorial brain, and spinal cord is required. Very recently, the 2024 revisions of the McDonald criteria included the incorporation of the optic nerve as an additional anatomic location for the demonstration of dissemination in space [6]. Recommendations on standardized image acquisition and interpretation of brain and spinal cord lesions suggestive of MS aid with respect to correct lesion classification and applying diagnostic criteria [7, 8]. However, in the clinical routine setting, the correct image interpretation in terms of the anatomic location as well as lesion differentiation from important differential diagnoses—such as small vessel disease—remains challenging, which poses the risk of MS misdiagnosis [9, 10]. This concerns the detection of new inflammatory disease activity during treatment monitoring but also the classification according to diagnostic criteria of patients presenting with a first clinical event suggestive of MS [11]. Studies on interrater agreement regarding the MRI analysis of patients at the stage of the initial presentation are limited and date back more than 15 years using MR systems operating at lower magnetic field strengths (≤ 1.5 Tesla) and 2‐dimensional (2D) image acquisition techniques with rather low spatial resolution parameters [12, 13, 14, 15, 16]. Overall, these studies showed a fair‐to‐moderate interobserver agreement regarding specific anatomic locations of DIS and regarding the DIS and DIT classification in general. However, the interrater agreement on MS lesion detection and the potential impact on diagnosis according to the 2017 and 2024 revisions of the McDonald criteria applying state‐of‐the‐art image acquisition techniques as recommended by the MAGNIMS‐CMSC‐NAIMS recommendations have not been investigated so far [7].

The aim of this study was to assess the interrater agreement regarding the detection and classification of MRI lesions in the brain, optic nerve, and spinal cord in patients presenting with a first clinical episode suggestive of MS. In addition, we tested the impact of interrater variability on the classification according to different diagnostic criteria such as the 2017 and the 2024 revisions of the McDonald criteria.

## 2 Methods

### 2.1 Study Design and Patient Selection

We prospectively included patients presenting with the first clinical episode suggestive of MS as a part of the so‐called “early MS cohort.” Inclusion criteria were: age between 18 and 65 years, first clinical event suggestive of chronic inflammatory demyelination. Exclusion criteria were defined as: past medical history of other inflammatory, vascular, or neoplastic disease of the central nervous system (CNS), pregnancy, contraindications regarding magnetic resonance imaging, and insufficient image quality.

This study was conceived as an exploratory interrater agreement analysis; therefore, no formal a priori sample size calculation was performed. The chosen cohort size (*n* = 78) is consistent with or larger than comparable interrater reliability studies in the field and provides robust data for κ‐statistic‐based agreement analyses.

### 2.2 MRI Acquisition Protocol

Image acquisition included a multisequence MRI protocol of the brain, the optic nerves, and spinal cord on a whole‐body MR system operating at 3 Tesla (Skyra, Siemens Healthineers, Erlangen, Germany). We used a 20‐channel head coil and the spine receive coil (Siemens Healthineers).

Brain MRI acquisition included sagittal 3‐dimensional (3D) fluid‐attenuated‐inversion‐recovery (FLAIR), axial T2‐weighted turbo/fast spin echo (TSE), and after the administration of a standard single dose (0.1 mmol/kg/bodyweight) intravenous Gadolinium‐based contrast media (Gd‐DOTA, Dotarem, Guerbert, Roissy, France) axial T1‐weighted turbo spin echo. Optic nerve imaging included axial and coronal fat‐saturated 2D T2‐weighted TSE and coronal contrast‐enhanced 2D fat‐saturated T1‐weighted turbo spin echo sequence. The spinal cord MRI image acquisition included the entire spinal cord applying the following pulse sequences: sagittal 2D short tau inversion recovery (STIR), sagittal 2D T2‐weighted TSE, and contrast‐enhanced sagittal 2D T1‐weighted TSE sequence. Details on the image acquisition protocol are summarized in Supplementary Tables 1–3. The image acquisition of the brain and the spinal cord has been performed according to the 2021 MAGNIMS‐CMSC‐NAIMS consensus recommendations on the use of MRI in MS [7].

### 2.3 Image Analysis and Interpretation

All images were presented on a dedicated workstation running Visage 7 client software (Visage 7.1, Visage Imaging Inc., San Diego, CA). The image analysis was performed by three readers with different degrees of expertise: a neuroradiologist with 20 years of experience (rater 1), a neuroradiologist with 10 years of experience (rater 2), and a radiology resident with 5 years of experience (rater 3). All readers were blinded to clinical presentation and paraclinical test results. Focal lesions in the brain, optic nerve, and spinal cord were considered and classified according to recent consensus expert panel recommendations [8, 17].

The interpretation of the brain regarding T2 lesions was based on a combined analysis of the 3D FLAIR images and axial T2‐weighted TSE; the assessment of T2 lesions in the spinal cord was based on a combined analysis of the sagittal 2D STIR and sagittal 2D T2‐weighted TSE (Figure 1a), and the assessment of T2 lesions in the optic nerves was based on a combined analysis of the axial and coronal fat‐saturated 2D T2‐weighted TSE (Figure 1b). Lesions were categorized according to the following anatomic locations: cortical gray matter/juxtacortical white matter (WM), periventricular WM, deep WM, infratentorial brain region, and spinal cord, as defined by the 2017 revisions of the McDonald criteria [5]. Regarding the 2024 McDonald criteria, the optic nerve (ON) was considered a fifth anatomic location for the demonstration of dissemination in space [6]. Although our MRI protocol included sequences sensitive to paramagnetic rim lesions and the central vein sign, these markers were not incorporated into the classification process, as they are not yet standardized for routine clinical application and were not part of the established diagnostic framework at the time of study design.

**FIGURE 1a:**
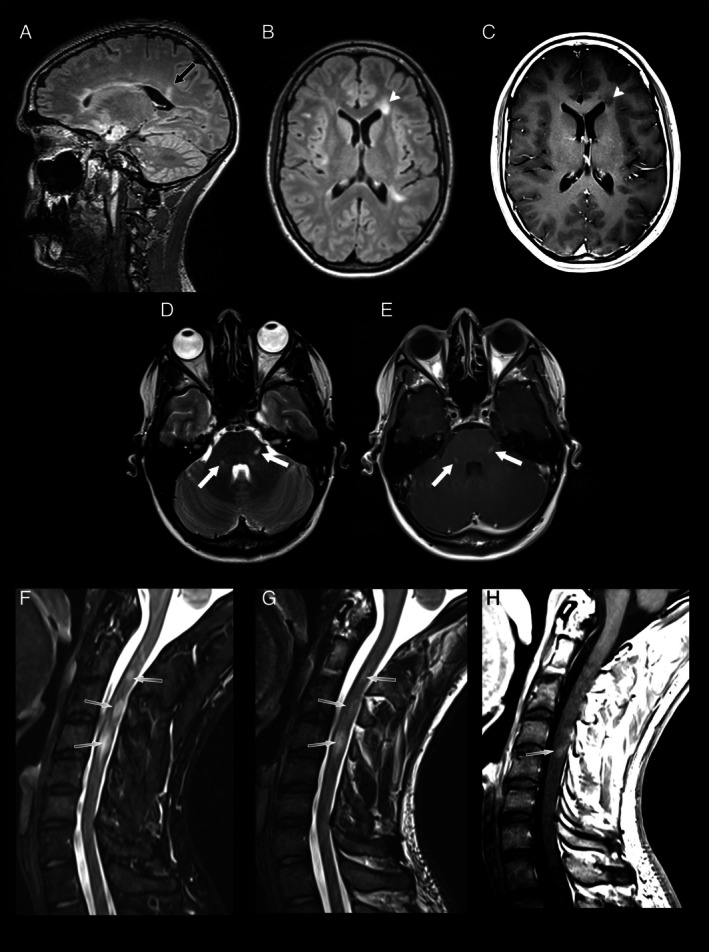
The image acquisition protocol followed the MAGNIMS‐CMSC‐NAIMS recommendations and included a (A) 3D FLAIR sequence with (B) multiplanar reconstruction, an axial T1 TSE (C and E) after contrast media injection, an (D) axial T2 TSE for brain imaging as well as a (F) sagittal T2 TSE STIR, (G) T2 TSE, and (H) T1 TSE after contrast media injection in separate acquisitions for the cervical as well as the thoracic (not shown) spine. In this 24‐year‐old female patient, periventricular lesions (black arrow) with partially lowered T1 signal (arrow heads) as well as infratentorial lesions showing contrast enhancement (white arrows) as well as lesions in the cervical spinal cord (gray arrows), in part with contrast enhancement were observed. Discrepancies between different readers were mainly due to different interpretations of lesions as being in the deep white matter or in juxtacortical or periventricular location.

**FIGURE 1b:**
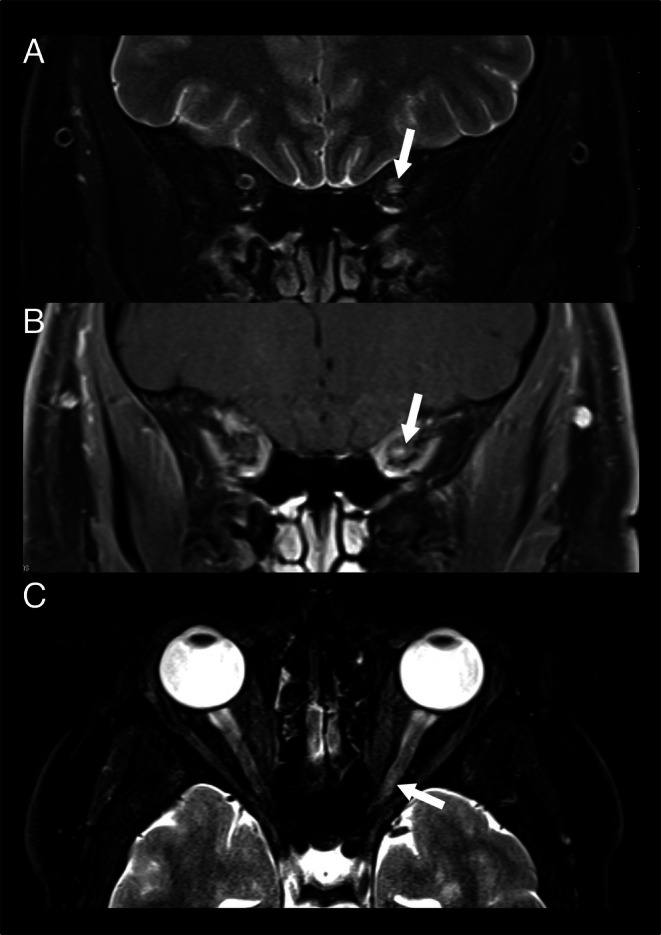
Optic nerve imaging included a (A) coronal T2 TSE with fat saturation, a (B) coronal T1 TSE with fat saturation as well as a (C) axial T2 TSE with fat saturation. In this 26‐year‐old male patient, a clinically acute optic neuritis was confirmed with MR imaging (arrows). In other cases, the signal alterations within the optic nerve were only subtle, leading to different interpretations by the readers.

The exact number of T2 and Gd‐enhancing lesions in each anatomic category was reported up to a number of 10 lesions. A lesion load higher than 10 lesions was reported as lesion number > 10. Based on the lesion count, each patient was classified according to the 2017 and 2024 revisions of the McDonald criteria and the 2016 MAGNIMS criteria [5, 6, 18]. In this process, DIS was defined by the presence of lesions in ≥ 2 anatomical locations, and DIT was defined by the presence of simultaneous Gd‐enhancing and non‐enhancing lesions. The final “diagnosis” variable used for interrater analysis was thus directly derived from the individual assessments of DIS and DIT.

### 2.4 Statistical Analysis

The interrater variability was calculated by Cohen's κ for each pair of readers and Fleiss's κ for the overall degree of agreement of all readers. κ resembles the degree of agreement, and can take values from −1 to 1, which can be interpreted as follows: ≤ 0,09 = agreement equivalent to chance; 0.10–0.20 = slight agreement; 0.21–0.40 = fair agreement; 0.41–0.60 = moderate agreement; 0.61–0.80 = substantial agreement; 0.81–0.99 = near‐perfect agreement; and 1.00 = perfect agreement. Negative κ values indicate that the observed concordance is inferior to what would be anticipated by chance [19]. Confidence intervals are presented alongside the *κ* values. Statistical significance was assumed with *p* ≤ 0.05. Statistical analyses were performed with SPSS (SPSS IBM, Armonk, NY, USA, Version 28).

## 3 Results

### 3.1 Patients

We prospectively included 78 patients. Patient characteristics are presented in Table 1. All patients underwent the complete neurological and neuroradiological diagnostic workup.

**TABLE 1:** Patient characteristics.

Number of patients	78
Sex (male/female)	28/50
Age median [range]	32 years [18–65]
Disease duration median*[range]	28 days [3;403]
Clinical presentation	
Symptoms of optic neuritis^a^	33 (42%)
Spinal cord syndrome^a^	13 (17%)
Polysymptomatic^a^	12 (15%)
Hemispheric syndrome^a^	10 (13%)
Brain stem syndrome^a^	10 (13%)

*Note:* *defined as time interval between first clinical presentation and MRI acquisition; ^a^ Data represent number of patients.

Abbreviation: CSF, cerebrospinal fluid.

### 3.2 Image Analysis

#### 3.2.1 Interrater Agreement Regarding Focal T2 and Gd‐Enhancing Lesions

**FIGURE 2:**
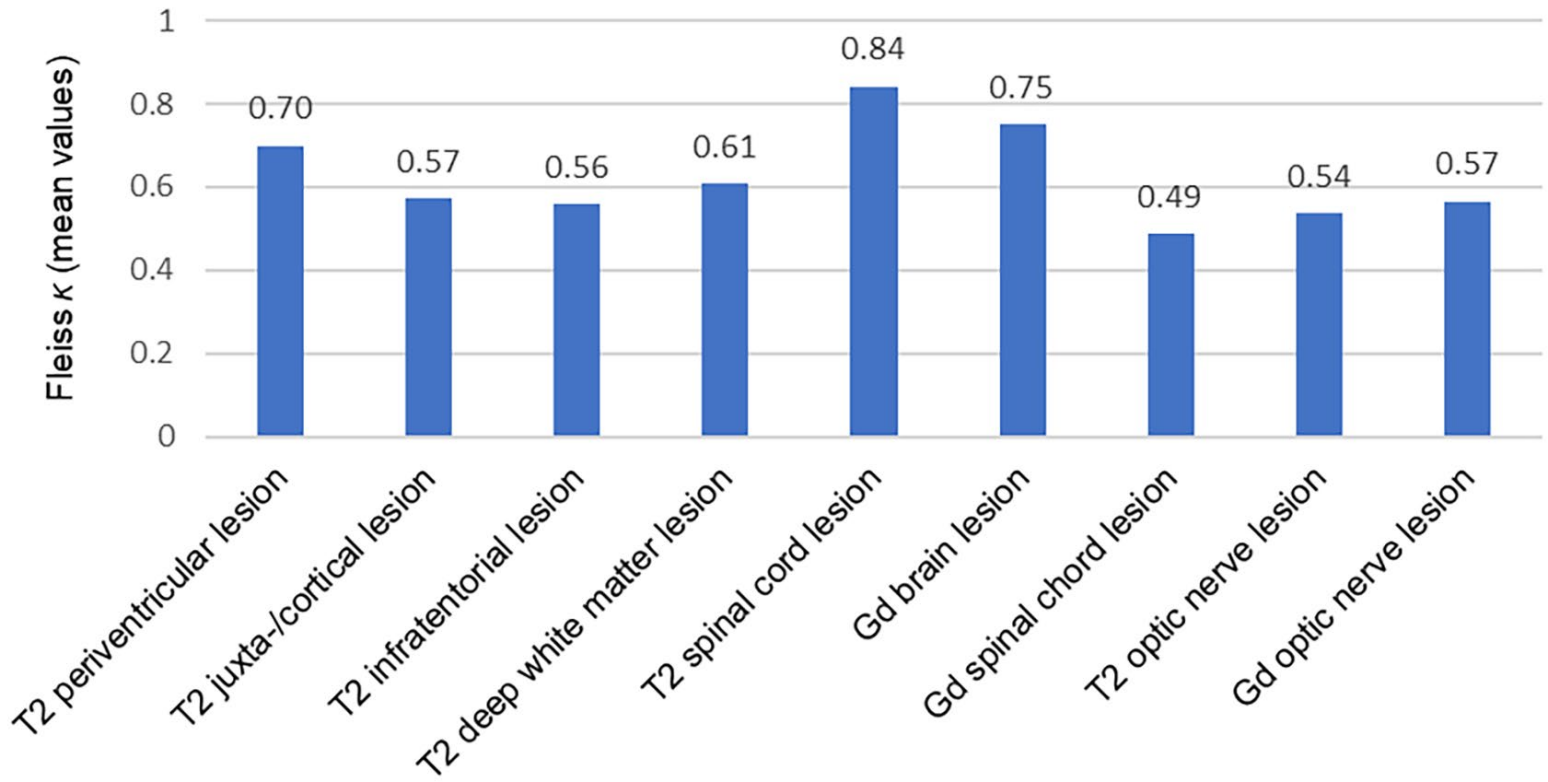
Fleiss κ Interrater agreement regarding brain, optic nerve, and spinal cord lesions with confidence intervals. Gd = Gadolinium.

Figure 2 summarizes the different levels of Fleiss κ interrater agreement regarding the presence of brain, optic nerve, and spinal cord lesions. The best interrater agreements were observed in the assessment of T2 lesions in the spinal cord (*κ* = 0.84) and Gd‐enhancing lesions in the brain (*κ* = 0.75). Considering different lesion locations in the brain, the highest interrater agreement was observed for periventricular lesions (*κ* = 0.70), while the lowest agreement was observed for infratentorial lesions (*κ* = 0.56). Moderate agreement was observed for T2 lesions (*κ* = 0.54) and Gd‐enhancing lesions (*κ* = 0.57) in the optic nerve. The results of the interrater agreement regarding the number of lesions in the brain and spinal cord are presented in Table 2. While the number of T2 juxtacortical and T2 periventricular lesions shows the lowest overall agreement (*κ* = 0.28–0.32), the number of Gd lesions and T2 spinal cord lesions has the best overall agreement (*κ* = 0.50–0.56).

**TABLE 2:** Interrater agreement regarding the number of lesions in the brain and spinal cord.

Criterion	Average number of lesions	Fleiss *κ*	Standard error
Number of T2 periventricular lesions	6	0.33	0.02
Number of T2 juxta−/cortical lesions	3	0.28	0.03
Number of T2 infratentorial lesions	2	0.40	0.03
Number of T2 deep white matter lesions	6	0.38	0.03
Number of T2 spinal cord lesions	2	0.56	0.03
Number of Gd spinal cord lesions	0	0.50	0.06
Number of Gd brain lesions	1	0.48	0.04

Abbreviation: Gd, Gadolinium.

**FIGURE 3:**
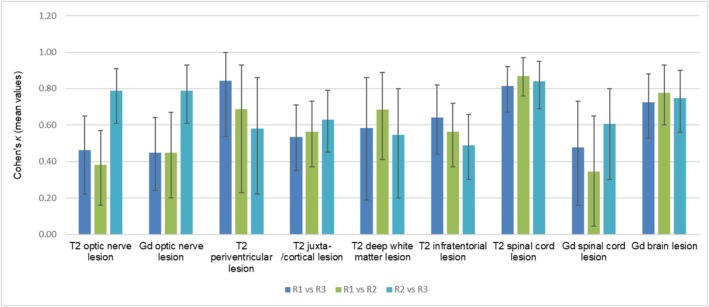
Cohen's κ interrater analysis for comparing different raters with confidence intervals. Gd = Gadolinium; *R* = rater.

The results from the Cohen's κ analysis highlighting the interrater agreement among the three raters are presented in Figure 3.

The evaluation of T2 ON lesions between raters 1 and 3 as well as raters 1 and 2 shows a fair‐to‐moderate agreement with *κ* values from 0.38 to 0.46 and 0.45 for Gd ON lesions, while the agreement between raters 2 and 3 shows substantial results for T2 and Gd ON lesions (0.79).

For periventricular and juxtacortical lesions, all rater pairs show moderate‐to‐substantial agreement, with *κ* values 0.65–0.84 (periventricular) and 0.53–0.63 (juxtacortical). Similar trends are observed for deep WM (*κ* = 0.55–0.68) and infratentorial lesions (*κ* = 0.49–0.64), where agreement among the raters remains substantial across most comparisons. For T2 spinal cord lesions, the raters show near perfect agreement (0.82–0.87), while Gd spinal cord lesions exhibit lower κ values (*κ* = 0.34–0.61).

Finally, for Gd brain lesions, the interrater agreement is substantial across all rater comparisons (*κ* = 0.73–0.78).

#### 3.2.2 Interrater Agreement Regarding the Classification According to Diagnostic Criteria

**FIGURE 4:**
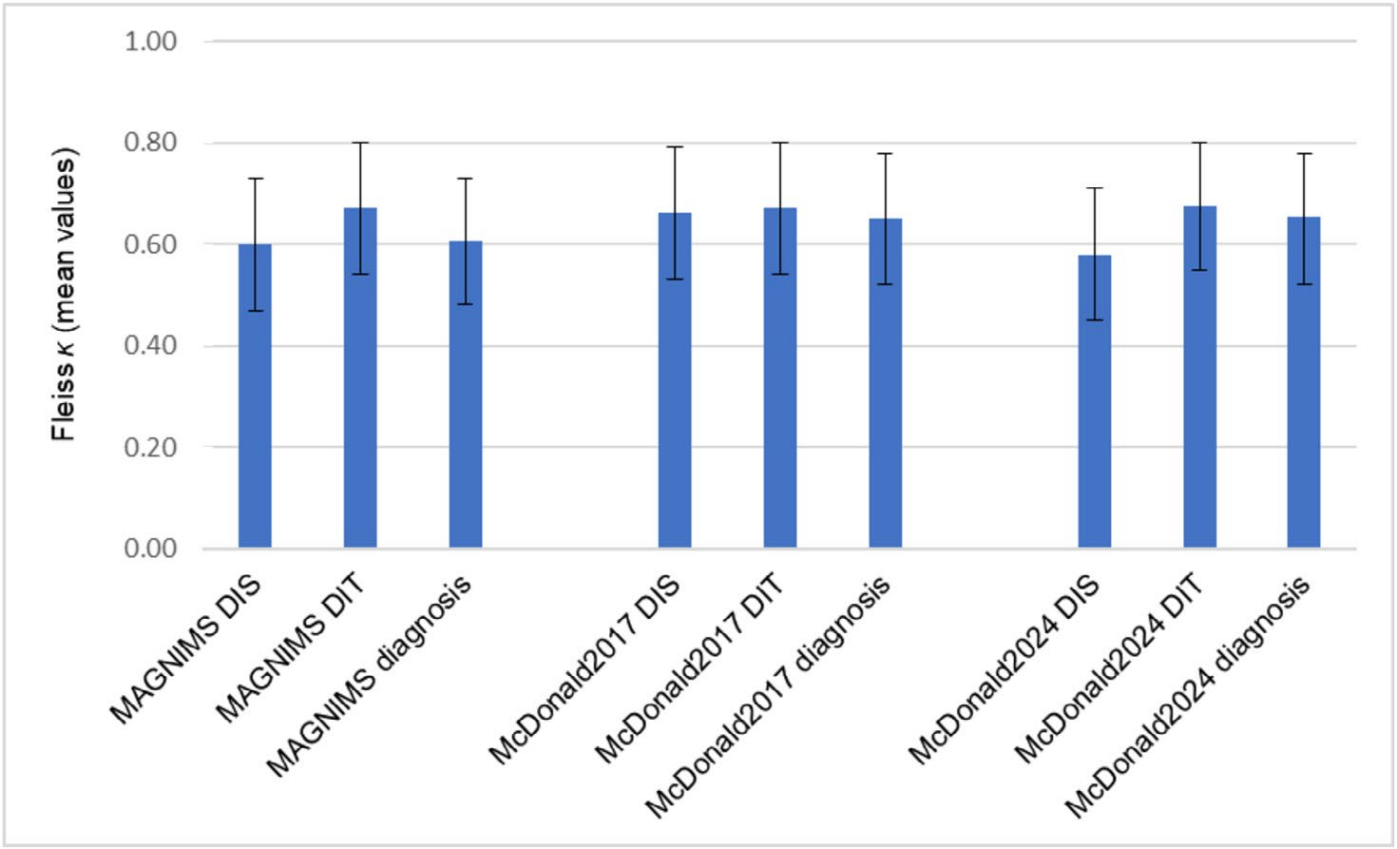
Fleiss K Interrater analysis concerning different diagnostic criteria with confidence intervals. DIT = Dissemination in time; DIS = Dissemination in space.

Figure 4 presents the levels of interrater agreement (Fleiss' Kappa analysis) regarding the classification according to different diagnostic criteria. The 2017 McDonald criteria demonstrated the best agreement for DIT and DIS, with overall agreement of 0.66 for DIS and 0.67 for DIT. However, all criteria exhibited moderate‐to‐substantial agreement across their respective dimensions (DIS = 0.58–0.66; DIT = 0.67; diagnosis = 0.61–0.65), indicating reliable classification frameworks with some variability in the assessment of DIT and DIS. The confidence intervals of the results are given in the graphs.

## 4 Discussion

The objective of this study was to evaluate interrater variability in MRI interpretation among patients experiencing an initial clinical episode indicative of multiple sclerosis.

Using a state‐of‐the‐art comprehensive and standardized MRI acquisition protocol of the brain, optic nerves, and spinal cord according to the MAGNIMS‐NAIMS‐CMSC recommendations, we obtained comparable results in terms of slight‐to‐fair interrater agreement regarding the assessment of the total number of lesions [7, 12]. Interestingly, the better image quality at 3 T and applying 3D FLAIR images did not lead to a higher diagnostic certainty with a positive impact on the interobserver variability. However, with respect to the clinically most relevant information, that is, whether one or more T2 lesions are present in a certain anatomic location according to diagnostic criteria, the interrater agreement reached moderate and substantial κ values (*κ* = 0.49–0.84). Overall, the interrater agreement in our study is slightly better than reported results obtained on datasets using lower magnetic field strengths (1.0 T), 2D image acquisitions, and rather lower spatial resolution (slice thickness of 5 mm) [14]. In particular, this is most obvious for periventricular and infratentorial lesions as well as for spinal cord lesions. Surprisingly, the interobserver agreement in our study regarding enhancing lesions was slightly inferior compared to the 2D datasets obtained at 1.0 T [14].

The assessment of lesions in the optic nerves on MRI is quite challenging in clinical practice. Despite the fact that optic neuritis is a quite frequent initial presentation of MS characterized by focal T2 hyperintense lesions that might show contrast enhancement, MRI lesions in the optic nerves were not incorporated into diagnostic criteria for a long time [1, 2, 3, 4, 5, 17]. Consequently, optic nerve MRI was not recommended to be used routinely in the diagnostic workup of patients presenting with clinical symptoms suggestive of MS [7]. Given the fact that the 2024 revisions of the McDonald criteria consider optic nerve involvement as a fifth anatomic category for the demonstration of DIS, it is important to know how reliable optic nerve lesions can be assessed on MRI. However, data on the reproducibility of optic nerve lesion assessment are limited. The image analysis of our dataset applying a standardized optic nerve MRI acquisition protocol according to recent international expert guidelines [7, 17, 20] resulted in moderate interrater agreement with respect to T2‐hyperintense (*κ* = 0.54) and contrast‐enhancing lesions (*κ* = 0.57) in the optic nerves. This rather high interrater variability compared to other regions such as periventricular and spinal cord lesions had probably a slight impact on the poorer interrater agreement with respect to the DIS according to the new revisions of the McDonald criteria compared to the 2017 revisions. A recent study applying 2D coronal short tau inversion recovery (STIR) images at 3 T showed a very low interrater agreement with an interclass correlation coefficient (ICC) of 0.054 [16]. The better agreement in our study might be due to the imaging acquisition in two anatomical planes and the use of fat‐saturated 2D T2‐weighted TSE.

Apparently, in this study, the interrater agreement with respect to the total number of lesions in different anatomical regions and the classification according to diagnostic criteria is not substantially positively influenced by advances in image acquisition, like the higher magnetic field strength and 3D acquisition techniques. Our agreement values are slightly better with respect to the lesion assessment in different anatomical regions but quite comparable on diagnostic criteria classification compared to data obtained at lower magnetic field strengths and 2D image acquisition [14]. This is in line with a multicenter MAGNIMS study showing that image analysis of images obtained at 3 T compared to 1.5 T did not lead to improved classification of patients according to McDonald criteria.

Beyond the image acquisition parameters, it is crucial to gain expertise among radiologists and neurologists in applying diagnostic criteria on MRI data of the brain and spinal cord in individual patients. Recent studies using questionnaires and involving American and European neurologists showed a need for improvement in image reading skills regarding the application of the 2017 revisions of the McDonald criteria [11, 21]. It can be anticipated that this will be even more important regarding the application of the new revisions of the McDonald criteria involving the optic nerve, paramagnetic rim lesions, and the assessment of the central vein sign [6].

In conclusion, even using a standardized state‐of‐the‐art image acquisition protocol and comparing raters with substantial expertise, the interrater agreement on the exact number of lesions in different anatomical locations remains low. However, good agreement values regarding the patient‐wise identification of lesions in anatomical locations incorporated into McDonald diagnostic criteria result in a good interrater agreement with respect to the classification according to the 2017 and new revisions of the diagnostic criteria. Even a slightly lower agreement regarding optic nerve lesions compared to other anatomical locations did not substantially impair the interrater agreement for DIS classification.

## Author Contributions

**Nima Mahmoudi:** conceptualization, investigation, Data curation, formal analysis, visualization writing – original draft. **Julius Renne:** conceptualization, investigation, writing – original draft. **Franz Felix Konen:** data curation, writing – Review and Editing. **Konstantin Fritz Jendretzky:** data curation, writing – Review and Editing. **Nora Möhn:** data curation, writing – Review and Editing. **Lea Grote‐Levi:** data curation, writing – Review and Editing. **Stefan Gingele:** data curation, writing – Review and Editing. **Martin W. Hümmert:** data curation, writing – Review and Editing. **Philipp Schwenkenbecher:** data curation, writing – Review and Editing. **Alexander Soldatov:** data curation, writing – Review and Editing. **Kurt‐Wolfram Sühs:** data curation, writing – Review and Editing. **Peter Raab:** writing – Review and Editing. **Thomas Skripuletz:** conceptualization, Data curation, writing – Review and Editing. **Mike P. Wattjes:** conceptualization, supervision, investigation, formal analysis, visualization, writing – original draft.

## Ethics Statement

The study protocol was approved by the local institutional review board (Hannover Medical School, Hannover, Germany, No. 8819_BO_S_2019) and was carried out according to the principles of the Declaration of Helsinki. Written informed consent was obtained from all patients prior to inclusion.

## Conflicts of Interest

**N. Mahmoudi** received travel compensation from Penumbra and Sanofi. **J. Renne** has nothing to disclose. **F. F. Konen** received travel compensation from Merck, Novartis, BMS, and Alexion and is a German Research Foundation (DFG)‐funded fellow as part of the Clinician Scientist Program (PRACTIS) at Hannover Medical School. **KF Jendretzky** received research support from Else Kröner Fresenius Foundation and travel compensation and congress fees from Merck, Neuraxpharm, and Novartis. **N. Möhn** received speaker honoraria from Alexion, Biogen, Novartis, and Merck as well as travel compensation from CLS Behring, Merck, and BMS. She is a German Research Foundation (DFG)‐funded fellow as part of the Clinician Scientist Program (PRACTIS) at Hannover Medical School. **L. Grote‐Levi** is a German Research Foundation (DFG)‐funded fellow as part of the Clinician Scientist Program (PRACTIS) at Hannover Medical School. **S. Gingele** reports research support from Alnylam Pharmaceuticals, CSL Behring, Else Kröner Fresenius Foundation, Deutsche Forschungsgemeinschaft, and Hannover Biomedical Research School (HBRS) and consulting and/or speaker honoraria from Alexion, Alnylam Pharmaceuticals, AstraZeneca, GSK, Pfizer, and Merck, all outside the submitted work. **M.W. Hümmert** received institutional research support from Myelitis e. V., German Federal Joint Committee/Innovation Fund, and NEMOS e. V.; speaker honoraria from selpers og, AMGEN/Horizon, and Alexion; and travel grants from Alexion and compensation for serving on an advisory board from Alexion, UCB, and Roche. **P. Schwenkenbecher** has nothing to disclose. **A. Soldatov** has nothing to disclose. **K.W. Sühs** reports honoraria for lectures or travel reimbursements for attending meetings from Biogen, Merck, Mylan, Roche, Bavarian Nordic, Viatris, and Bristol‐Myers Squibb, as well as research support from Bristol‐Myers Squibb. **P. Raab** reports honoraria for lectures from Biogen. **T. Skripuletz** reports research support from Alnylam Pharmaceuticals, CSL Behring, Novartis, Siemens; honoraria for lectures and travel expenses for attending meetings from Alexion, Alnylam Pharmaceuticals, argenx, Bayer Vital, Biogen, Bristol Myers Squibb, Celgene, Centogene, CSL Behring, Euroimmun, Grifols, Hexal AG, Horizon, Janssen‐Cilag, Merck Serono, Novartis, Pfizer, Roche, Sanofi, Siemens, Swedish Orphan Biovitrum, Teva, Viatris; consultant fees from Alexion, Alnylam Pharmaceuticals, Biogen, Centogene, CSL Behring, Grifols, Hexal AG, Janssen‐Cilag, Merck Serono, Novartis, Roche, Sanofi, Swedish Orphan Biovitrum, Viatris. **M.P. Wattjes** received speaker or consultancy honoraria from Bayer Healthcare, Biogen, Biologix, Bristol Myers Squibb, Celgene, Genilac, Imcyse, IXICO, Icometrix, Medison, Merck‐Serono, Novartis, Roche, Spinger Healthcare, Sanofi‐Genzyme.

## Supporting information


**Supplementary Table 1** Brain sequence parameters per pulse sequence.
*Note:* 3D‐T1w = 3 dimensional T1‐weighted, 2D‐T2w = 2 dimensional dual‐echo T2 weighted, FLAIR = fluid‐attenuated inversion recovery; TSE = turbo spin echo, TR = repetition time, TE = echo time, TI = inversion time.
**Supplementary Table 2** Orbit sequence parameters per pulse sequence.
*Note:* 2D‐T1w = 2 dimensional T1‐weighted, 2D‐T2w = 2 dimensional dual‐echo T2 weighted, TSE = turbo spin echo, TR = repetition time, TE = echo time.
**Supplementary Table 3** Spine sequence parameters per pulse sequence.
*Note:* 3D‐T1w = 3 dimensional T1‐weighted, 2D‐T2w = 2 dimensional dual‐echo T2 weighted; TSE = turbo spin echo, TR = repetition time, TE = echo time, TI = inversion time.

## Data Availability

The data that support the findings of this study are available on request from the corresponding author. The data are not publicly available due to privacy or ethical restrictions.
