# Supplementary material for: Does Interrater Variation Influence the Classification According to the Diagnostic Criteria for Multiple Sclerosis?
Source: Eur J Neurol. 2026 Jan 23;33(1):e70469. doi: 10.1111/ene.70469 (PMC12828338; doi:10.1111/ene.70469)
Supplement: Supplementary file 1 — Supplementary Table 1 Brain sequence parameters per pulse sequence. Note: 3D‐T1w = 3 dimensional T1‐weighted, 2D‐T2w = 2 dimensional dual‐echo T2 weighted, FLAIR = fluid‐attenuated inversion recovery; TSE = turbo spin echo, TR = repetition time, TE = echo time, TI = inversion time. Supplementary Table 2 Orbit sequence parameters per pulse sequence. Note: 2D‐T1w = 2 dimensional T1‐weighted, 2D‐T2w = 2 dimensional dual‐echo T2 weighted, TSE = turbo spin echo, TR = repetition time, TE = echo time. Supplementary Table 3 Spine sequence parameters per pulse sequence. Note: 3D‐T1w = 3 dimensional T1‐weighted, 2D‐T2w = 2 dimensional dual‐echo T2 weighted; TSE = turbo spin echo, TR = repetition time, TE = echo time, TI = inversion time. [file ENE-33-e70469-s001.docx]

**Supplementary tables**

**Supplementary Table 1. Brain sequence parameters per pulse sequence**

| Contrast | 3D-FLAIR | 2D-T2w | | 2D-T1 |  |
| --- | --- | --- | --- | --- | --- |
| Sequence | SPACE | | TSE | SE | |
| Anatomic orientation | sagittal | | axial | axial | |
| TR [ms] | 5000 | | 4200 | 489 | |
| TE [ms] | 388 | | 89 | 9.2 | |
| TI 1 [ms] | 1800 | | - | - | |
| TI 2 [ms] | - | | - | - | |
| Flip Angle 1 [°] | variable | | 150 | 70 | |
| Flip Angle 2 [°] |  | | - | 180 | |
| Turbo Factor [-] | 278 | | 18 | - | |
| Fat suppression | - | | - | - | |
| FOV [mm^3^] (APxFHxRL) | 260x260x173 | | 240x135x240 | 263x350x350 | |
| Spatial Resolution [mm^3^] | 0.5x0.5x0-9 | | 0.6x0.6x3.0 (axial) | 0.6x0.6x3.0 (axial) | |
| Acquisition time [min:sec] | 5:47 | | 2:08 | 3:11 | |

**Note:** 3D-T1w = 3 dimensional T1-weighted, 2D-T2w = 2 dimensional dual-echo T2 weighted, FLAIR = fluid-attenuated inversion recovery; TSE = turbo spin echo, TR = repetition time, TE = echo time, TI = inversion time

**Supplementary Table 2. Orbit sequence parameters per pulse sequence**

| Contrast | 2D-T2w | | 2D-T2w | 2D-T1w | |  |
| --- | --- | --- | --- | --- | --- | --- |
| Sequence | | TSE | TSE | | TSE | |
| Anatomic orientation | | axial | coronal | | coronal | |
| TR [ms] | | 6920 | 3650 | | 590 | |
| TE [ms] | | 84 | 84 | | 9 | |
| Flip Angle 1 [°] | | 150 | 150 | | 150 | |
| Turbo Factor [-] | | 18 | 18 | | 3 | |
| Fat suppression | | fatsat | SPAIR | | fatsat | |
| FOV [mm^3^] (APxFHxRL) | | 200x96x200 | 79x200x200 | | 79x200x200 | |
| Spatial Resolution [mm^3^] | | 0.4 x0.4 x 3.0 | 0.4x0.4x3.0 | | 0.6x0.6x3.0 | |
| Acquisition time [min:sec] | | 2:13 | 2:20 | | 3:11 | |

**Note:** 2D-T1w = 2 dimensional T1-weighted, 2D-T2w = 2 dimensional dual-echo T2 weighted, TSE = turbo spin echo, TR = repetition time, TE = echo time

**Supplementary Table 3. Spine sequence parameters per pulse sequence**

| Contrast | 2D-T2w | | 2D-T2w-STIR | 2D-T1w | | 2D-T2w | 2D-T2w-STIR | | | 2D-T1w |
| --- | --- | --- | --- | --- | --- | --- | --- | --- | --- | --- |
|  | cervical | | cervical | cervical | | thoracic | thoracic | | | thoracic |
| Sequence | TSE | TSE | | TSE | TSE | | | TSE | TSE | |
| Anatomic orientation | sagittal | sagittal | | sagittal | sagittal | | | sagittal | sagittal | |
| TR [ms] | 3500 | 3700 | | 450 | 3500 | | | 3700 | 450 | |
| TE [ms] | 104 | 46 | | 9.5 | 100 | | | 44 | 8.7 | |
| TI 1 [ms] | - | 230 | | - | - | | | - | - | |
| Flip Angle 1 [°] | 160 | 160 | | 150 | 140 | | | 160 | 150 | |
| Turbo Factor [-] | 21 | 15 | | 4 | 19 | | | 15 | 3 | |
| Fat suppression | - | STIR | | - | - | | | STIR | - | |
| FOV [mm^3^] (APxFHxRL) | 263x350x350 | 263x350x350 | | 263x350x350 | 263x350x350 | | | 263x350x350 | 263x350x350 | |
| Spatial Resolution [mm^3^] | 0.6 x0.6 x 3.0 (sagittal) | 0.4 x0.4 x 3.0 (sagittal) | | 0.6x0.6x3.0 (sagittal) | 0.7 x0.7 x 3.0 (sagittal) | | | 0.5x0.5x3.0 (sagittal) | 0.8x0.8x3.0 (sagittal) | |
| Acquisition time [min:sec] | 1:33 | 3:36 | | 1:49 | 1:57 | | | 3:36 | 2:50 | |

**Note:** 3D-T1w = 3 dimensional T1-weighted, 2D-T2w = 2 dimensional dual-echo T2 weighted; TSE = turbo spin echo, TR = repetition time, TE = echo time, TI = inversion time
